# COSMIN methodology for evaluating the content validity of patient-reported outcome measures: a Delphi study

**DOI:** 10.1007/s11136-018-1829-0

**Published:** 2018-03-17

**Authors:** C. B. Terwee, C. A. C. Prinsen, A. Chiarotto, M. J. Westerman, D. L. Patrick, J. Alonso, L. M. Bouter, H. C. W. de Vet, L. B. Mokkink

**Affiliations:** 1 Department of Epidemiology and Biostatistics and Amsterdam Public Health Research Institute, VU University Medical Center, P.O. Box 7057, 1007 MB Amsterdam, The Netherlands; 2 Department of Health Sciences and Amsterdam Public Health Research Institute, VU University, Amsterdam, The Netherlands; 3 Department of Health Services, University of Washington, Seattle, WA USA; 4 IMIM (Hospital del Mar Medical Research Institute), Department of Experimental and Health Sciences, Pompeu Fabra University (UPF), Barcelona, Spain; 5 CIBER en Epidemiología y Salud Pública (CIBERESP), Madrid, Spain; 6 Faculty of Humanities, Department of Philosophy, VU University, Amsterdam, The Netherlands

**Keywords:** Patient outcome assessment, Validation studies, Content validity, Patient-reported outcome, COSMIN, Systematic review

## Abstract

**Background:**

Content validity is the most important measurement property of a patient-reported outcome measure (PROM) and the most challenging to assess. Our aims were to: (1) develop standards for evaluating the quality of PROM development; (2) update the original COSMIN standards for assessing the quality of content validity studies of PROMs; (3) develop criteria for what constitutes good content validity of PROMs, and (4) develop a rating system for summarizing the evidence on a PROM’s content validity and grading the quality of the evidence in systematic reviews of PROMs.

**Methods:**

An online 4-round Delphi study was performed among 159 experts from 21 countries. Panelists rated the degree to which they (dis)agreed to proposed standards, criteria, and rating issues on 5-point rating scales (‘strongly disagree’ to ‘strongly agree’), and provided arguments for their ratings.

**Results:**

Discussion focused on sample size requirements, recording and field notes, transcribing cognitive interviews, and data coding. After four rounds, the required 67% consensus was reached on all standards, criteria, and rating issues. After pilot-testing, the steering committee made some final changes. Ten criteria for good content validity were defined regarding item relevance, appropriateness of response options and recall period, comprehensiveness, and comprehensibility of the PROM.

**Discussion:**

The consensus-based COSMIN methodology for content validity is more detailed, standardized, and transparent than earlier published guidelines, including the previous COSMIN standards. This methodology can contribute to the selection and use of high-quality PROMs in research and clinical practice.

**Electronic supplementary material:**

The online version of this article (10.1007/s11136-018-1829-0) contains supplementary material, which is available to authorized users.

## Introduction

Content validity is the degree to which the content of an instrument is an adequate reflection of the construct to be measured [1]. It refers to the relevance, comprehensiveness, and comprehensibility of the PROM for the construct, target population, and context of use of interest. It is often considered to be the most important measurement property of a patient-reported outcome measure (PROM). Messick emphasized the importance of content relevance and coverage for educational tests to determine what students have learned from a course. Each item on the test should relate to one of the course objectives, and each part of the course should be represented by one or more questions [2]. The same principles apply to the content of a PROM. All items in a PROM should be relevant for the construct of interest (within a specific population and context of use) and the PROM should be comprehensive with respect to patient concerns [3–7]. Furthermore, the PROM should be understood by patients as intended. The importance of content validity is stressed by the US Food and Drug Administration (FDA) [8] and the European Medicines Agency [9].

Lack of content validity can affect all other measurement properties. Irrelevant items may decrease internal consistency, structural validity, and interpretability of the PROM. Missing concepts may decrease validity and responsiveness. A high Cronbach’s alpha is no guarantee that the construct of interest is being measured or that no important concepts are missing [10, 11], and a high test–retest reliability or responsiveness does not imply that all items are relevant and that no important concepts are missing. One may measure the incomplete or incorrect construct very reliably and a real change in the construct of interest may be over- or underestimated due to irrelevant or missing concepts. Moreover, patients might become frustrated when questions that appear irrelevant to them are asked or when important questions are not asked, which may lead to biased responses or low response rates [3, 12].

The FDA guidance on patient-reported outcomes recommends to establish content validity before evaluating other measurement properties [8]. Also the consensus-based standards for the selection of health measurement instruments (COSMIN) initiative recommends to consider content validity first when evaluating and comparing measurement properties of PROMs in a systematic review [13]. In a recent international Delphi study on the selection of outcome measurement instruments for a core outcome set (COS), consensus was reached that at least content validity and internal structure should be adequate for recommending an instrument for a COS [14].

It is not easy to assess whether a PROM has good content validity. Many PROMs intend to measure complex and unobservable concepts, such as depression or fatigue. It is not straightforward to decide whether the construct is clear, whether all items are relevant, and whether the PROM is comprehensive. For example, does the item ‘I have energy’ belong in a PROM measuring fatigue (as a positively worded item) or does it measure a (slightly) different construct such as vitality? When asked, patients will typically come up with items that they consider to be missing, but are these really key aspects of the construct or are these variations of concepts already included or aspects of other constructs?

A well-designed PROM development study helps to ensure content validity [15–17]. Guidelines exist for performing qualitative studies to obtain patient input for good content coverage [4–6, 8, 15]. However, no guidelines exist for evaluating the quality of PROM development in a comprehensive and quantitative way.

Content validity of existing PROMs can be assessed by asking patients and professionals about the relevance, comprehensiveness and comprehensibility of the items, response options, and instructions [3, 18]. However, the methods used vary widely and many studies only address comprehensibility without paying attention to relevance and comprehensiveness [19]. Guidelines are needed for assessing the methodological quality of content validity studies. The COSMIN checklist was developed for assessing the methodological quality of studies on measurement properties and consists of nine boxes, containing standards (design requirements and preferred statistical methods) for assessing the methodological quality of studies; one box per measurement property [20]. The box on content validity needs to be updated for three reasons: first, the box does not contain standards for evaluating the quality of PROM development; second, no attention is paid to the comprehensibility of the PROM; third, the standards only concern whether certain things were done, but not how they were done (e.g., no standards were included for how it should be assessed whether all items are relevant).

In addition, criteria are needed for what constitutes good content validity to provide transparent and evidence-based recommendations for the selection of PROMs in systematic reviews, to determine whether a PROM is good enough to measure outcomes for regulatory approval of a new drug, or for inclusion in a PROM registry. The content validity of PROMs can be rated using review criteria of the Scientific Advisory Committee of the Medical Outcomes Trust (MOT) [21, 22], the evaluating the measurement of patient-reported outcomes (EMPRO) tool [23], the criteria published by Terwee et al. [24], or the minimum standards recommended by the International Society for Quality of Life Research (ISOQOL) [25]. However, these criteria are all only broadly defined and include no specific criteria for rating the relevance, comprehensiveness and comprehensibility of a PROM in a standardized way. Also, no methods exist yet for combining the evidence from the PROM development study and additional content validity studies in a systematic review. Grading of recommendations assessment, development, and evaluation (GRADE) offers a transparent and structured process for grading the quality of the evidence in systematic reviews of intervention studies [26], and can be used for developing a comparable methodology for evaluating the content validity of PROMs.

Our aims were to: (1) develop standards for evaluating the quality of PROM development; (2) update the original COSMIN standards for assessing the quality of content validity studies of PROMs; (3) develop criteria for what constitutes good content validity of PROMs, and (4) develop a rating system for summarizing the evidence on a PROM’s content validity and grading the quality of the evidence in systematic reviews of PROMs.

## Methods

### Study design

An international Delphi study of three online surveys was planned among a panel of experts. A fourth round was added to discuss six minor changes. The Delphi study was carried out by the day-to-day project team (CT, CP, LM, HV), in close collaboration with the steering committee (consisting of all authors). The steering committee discussed the draft Delphi questionnaires and all versions of the manuscript and made final decisions in the case that consensus had not been reached by the Delphi panel and when issues came up in the pilot testing after the Delphi study. In each round, panelists were asked to rate the degree to which they (dis)agreed to proposed standards and criteria on a 5-point rating scale (‘strongly disagree’ to ‘strongly agree’), and provide arguments for their ratings. If participants felt unqualified to answer a specific question, they could choose the response option ‘no opinion.’

### Literature search

Proposed standards and criteria were based on three literature searches: (1) a search used for developing the ISOQOL minimum standards for PROMs [25]; (2) a search on methods for selecting outcome measurement instruments for COS [14]; and (3) a PubMed search “content validity”[ti]. In addition, relevant text books and articles were used (e.g., International Society For Pharmacoeconomics and Outcomes Research (ISPOR) taskforce papers [4, 5], patient-reported outcome measurement information system (PROMIS) standards [27], and British Medical Journal (BMJ) guidelines for qualitative research [28]).

### Panelists

We intended to include participants with different areas of expertise, such as qualitative research, PROM development and evaluation, and systematic reviews of PROMs, and different professional backgrounds, such as clinicians, psychometricians, epidemiologists, and statisticians. We invited the 43 panelists of the original COSMIN Delphi study [20], 101 authors who used the COSMIN checklist (identified in the COSMIN database of systematic reviews [29] and by a PubMed search (COSMIN[tiab] OR “Consensus-based standards” [tiab])), 129 COSMIN users who corresponded with the COSMIN group, corresponding authors of 25 methodological papers on content validity and 64 content validity studies (identified by a PubMed search “content validity”[ti]), and 25 experts in qualitative research or PROM validation (identified by the authors). In total, we invited 340 people and aimed to include about 100 panelists. Information of the panelists was collected in round 1 regarding country, professional background, experience in qualitative research, and experience with PROM development, evaluation, and systematic reviews of PROMs.

### Delphi study

**Fig. 1:**
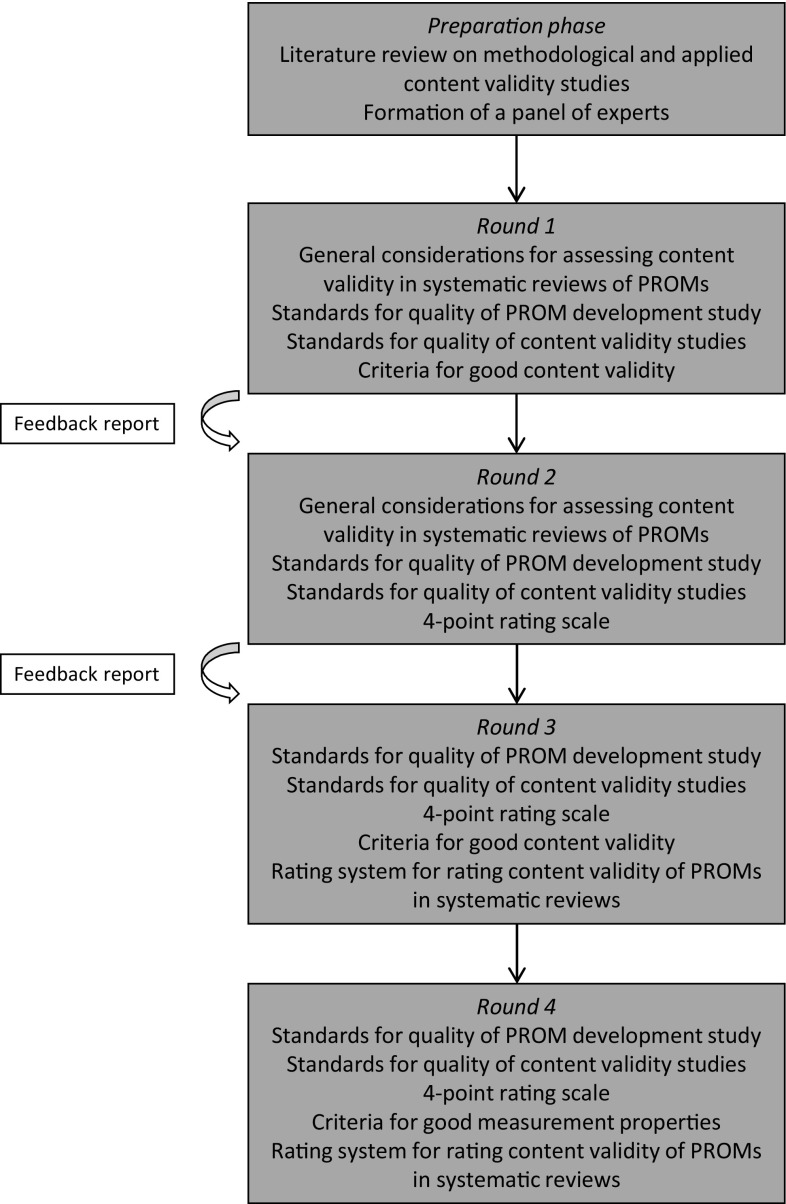
Design of the Delphi study

In round 1 (Fig. 1), general recommendations on performing a systematic review on content validity of PROMs were discussed (e.g., required expertise, scope of the review). In addition, two sets of standards (design requirements) were discussed: (1) standards for evaluating the quality of PROM development; and (2) standards for evaluating the quality of content validity studies of existing PROMs. Standards were presented as questions, similar to the original COSMIN standards [20]. In round 2, a 4-point rating scale was proposed for each standard. In round 1 and 3, criteria for what constitutes good content validity were discussed and how they should be rated per study. In round 3, a rating system was discussed for summarizing the evidence on a PROM’s content validity in systematic reviews of PROMs. Proposals were discussed for how an overall content validity rating per PROM can be determined. Finally, a proposal was discussed for grading the quality of the total body of evidence on a PROM, based on GRADE [26], taking into account study design, study quality, consistency and directness of study results, and the reviewer’s rating. An additional fourth round was needed to discuss six minor changes in the standards and criteria, based on the comments provided by the panellists in round 3.

A feedback report was provided in round 2 and 3, including response percentages and arguments to all questions of the previous round. In round 4, feedback of round 3 was considered not necessary because only six issues were discussed.

### Analyses

All results were analyzed anonymously. Consensus was considered to be reached when at least 67% of the panelists (strongly) agreed with a proposal. When consensus was not reached, a modified proposal was discussed in the next round. When strong arguments were provided against a proposal, even though consensus was reached, the steering committee decided whether it was necessary to propose an alternative in the next round.

### Pilot-testing

The pre-final standards, criteria, and rating system were pilot-tested by five authors (CB, CP, AC, HV, and LM) in a systematic review of PROMs measuring physical functioning in patients with low back pain [19] and in a systematic review of PROMs for hand osteoarthritis (manuscript in preparation). Issues that came up during the pilot test were discussed within the steering committee, resulting in final changes in the standards, criteria, and rating system. The rating system was also discussed with the chairman of the Dutch GRADE network. Finally, the “COSMIN Methodology for assessing the content validity of PROMs—user manual” was written, available from http://www.cosmin.nl.

## Results

The number of panelists participating/invited per round were as follows: 158/340 (46%), 122/316 (39%), 84/307 (27%), and 69/84 (82%) in rounds 1–4, respectively. In rounds 1–3, all eligible panelists were invited (denominators vary because some people were unreachable during part of the study), while in round 4 only the panelists who responded to round 3 were invited because only six minor issues were discussed. In total, 159 panelists from 21 countries participated (Table 1**)**.

**Table 1:** Characteristics of the respondents to each round of the Delphi study

	Round 1	Round 2	Round 3	Round 4
Number of participants	158	122	84	69
Country/region (*n*)				
US	24	21	18	13
Canada	15	7	6	6
UK	30	20	14	11
Netherlands	25	18	8	6
Europe other (11 countries)	37	32	24	21
Australia/New Zealand	16	12	9	8
Asia	3	1	1	1
Middle East	1	1	1	1
South America	1	1	1	1
Unknown	6	9	2	1
Professional background (*n*)^a^				
Allied health care professional	69	38	32	29
Medical doctor	19	9	4	4
Clinimetrician/psychometrician	33	19	15	12
Epidemiologist	30	19	13	12
Statistician	6	2	2	2
Other	54	27	23	19
Unknown		36	16	9
Current professional activity (*n*)^a^				
Clinician	35	16	12	10
Researcher	146	81	64	57
Journal editor	8	6	4	3
Other	27	14	12	12
Unknown		36	16	9
Experience in qualitative research				
A lot-some/a little-none (%)	65/35	67/33	71/29	70/30
Unknown (*n*)		36	16	9
Experience in development of PROMs				
A lot-some/a little-none (%)	58/42	60/40	66/34	61/39
Unknown (*n*)		37	17	10
Experience in evaluation of measurement properties of PROMs				
A lot-some/a little-none (%)	85/15	92/8	90/10	90/10
Unknown (*n*)		37	17	10
Experience in evaluation of content validity of PROMs				
A lot-some/a little-none (%)	75/25	76/24	79/21	75/25
Unknown (*n*)		36	16	9
Experience in systematic reviews of PROMs	70/30	72/28	72/28	70/30
A lot-some/a little-none (%)		36	16	9
Unknown (*n*)				
Ever used the COSMIN checklist				
Yes/no (%)	82/18	80/20	79/21	82/18
Unknown (*n*)		36	16	9

*PROMs* patient-reported outcome measures

^a^Multiple responses allowed

The number of issues discussed ranged from 78 in round 1 to six in round 4. In round 1, consensus was reached on 65/78 (82%) issues. The required 67% consensus was reached on all issues after round 4 (Table 2). Consensus was reached on four general recommendations on performing a systematic review on content validity of PROMs (Table 3).

**Table 2:** Number of issues on which consensus was reached in relation to the number of issues discussed in each round

Topic	Round 1	Round 2	Round 3	Round 4
General considerations in the evaluation of content validity of PROMS in systematic reviews of PROMs	6/8	2/2	NA	NA
Standards for evaluating the methodological quality of studies on the development of a PROM (box 1)				
Standards for evaluating the methodological quality of qualitative research performed to identify relevant items for a new PROM (box 1, part 1)	14/20	30/30^a^	3/3	2/2
Standards for evaluating the quality of a cognitive interview study performed to evaluate comprehensibility and comprehensiveness of a PROM (box 1, part 2)	11/14	25/25^a^	NA	NA
Standards for evaluating the quality of studies on content validity of PROMs (box 2)				
Standards for asking patients to rate the relevance, comprehensiveness, and comprehensibility of the items for the population of interest (box 2 part 1)	8/8	9/9	1/1	NA
Standards for asking professionals to rate the relevance of the items for the construct of interest (box 2 part 2)	6/7	6/6	NA	NA
Criteria for what constitutes good content validity of PROMs	20/21	NA	7/8	1/1
Rating system for rating the content validity of PROMs in a systematic review	NA	NA	6/6	3/3
Total	65/78 (82%)	71/71 (100%)	17/18 (94%)	6/6 (100%)

*NA* Not applicable (not discussed in the round)

^a^New issues concerned the 4-point rating scale for the standards

**Table 3:** General recommendation on how to perform a systematic review on the content validity of PROMs

Authors of a systematic review of PROMs should clearly define the scope^a^ of their review. This scope should be the reference point for evaluating content validity of the included PROMs
Content validity should be evaluated by at least two reviewers, independently
We recommend that the review team includes reviewers with at least some knowledge of the construct of interest; experience with the target population of interest; and some knowledge or experience with PROM development and evaluation, including qualitative research
The review team should also consider the content of the PROMs themselves

See Prinsen et al. for further details [13]

^a^By scope we mean the construct, target population, and measurement aim (e.g., evaluation) of interest in the review

### Standards for evaluating the quality of PROM development (COSMIN box 1, supplementary material A1)

The standards in this box are divided into two parts: Part 1 concerns standards for evaluating the quality of research performed to identify relevant items for a new PROM; Part 2 concerns standards for evaluating the quality of a cognitive interview study or other pilot test (e.g., a survey or a Delphi study) performed to evaluate comprehensiveness and comprehensibility of the PROM.

Part 1 (identify relevant items for the PROM): in round 1 consensus was reached on including 14 out of 20 proposed standards, referring to general design requirements of a PROM development study (e.g., clear description of the construct of interest, target population, and context of use (i.e., the application(s) the PROM was developed for, e.g., discrimination, evaluation, prediction, and the way the PROM is to be used), study performed in a sample representing the target population), and standards for concept elicitation (e.g., appropriate qualitative methods and data analysis). Consensus was not reached on sample size requirements. It was argued that saturation is more important than sample size. We did not reach consensus on whether field notes should be made during focus groups or interviews. This was considered not necessary if focus groups or interviews were recorded. Consensus was also not reached on returning transcripts to participants for comments or corrections. It was argued that this is not a gold standard practice. Finally, we did not reach consensus on whether a translatability review should be performed. It was argued that if a PROM will (later) be used in another population than for which it was developed, the content validity should be reevaluated in that new population. In round 2, consensus was reached on not including the six standards discussed above.

Part 2 (cognitive interview study): in round 1 consensus was reached on including 11 out of 14 proposed standards, referring to general design requirements of a cognitive interview study (e.g., each item tested in an appropriate number of patients, representing the target population), and standards for assessing comprehensiveness and comprehensibility (e.g., appropriate cognitive debriefing methods, problems regarding comprehensibility appropriately addressed). Consensus was not reached in round 1 on including three standards on recording, field notes, and transcribing cognitive interviews. It was argued that recording is less important in this phase, as opposed to the item development phase. However, other panelists argued that it is important in this stage to record facial expressions, puzzlement, etc. The ISPOR recommendations [5] suggest recording and transcribing cognitive interviews for transparency reasons. In round 2 consensus was reached on including the standards on recording and transcribing but not including the standard on field notes because this was considered not essential if interviews were recorded.

In round 2, it was proposed to rate each standard on a 4-point rating scale, similar to the original COSMIN checklist [30]. In a related study on the COSMIN Risk of Bias checklist for PROMs [31], the COSMIN steering committee decided to rename the original labels excellent, good, fair, and poor into very good, adequate, doubtful, and inadequate, respectively. A total rating per box can be obtained by taking the lowest rating of any item in the box (‘worst score counts’) [30]. This method was chosen because poor methodological aspects of a study cannot be compensated by good aspects.

For all standards, consensus was reached on what constitutes a very good, adequate, doubtful, or inadequate rating. However, the rating scale of the standard on coding qualitative data was discussed again in rounds 3 and 4 because there were different opinions on the amount of data that need to be coded independently for getting a very good rating. Consensus was reached in round 4 that at least 50% of the data should be coded by at least two researchers independently for a very good rating.

During pilot testing, one change in this box was made by the steering committee: In the Delphi study, consensus was reached that comprehensiveness was not applicable for large item banks. However, during pilot testing members of the steering committee argued that item banks should also be comprehensive to patient concerns. The whole steering committee agreed and therefore the response option ‘not applicable because of large item bank’ was removed for standards on comprehensiveness, against the consensus reached in the Delphi study.

### Standards for evaluating the quality of content validity studies of PROMs (COSMIN box 2, supplementary material A2)

In round 1, consensus was reached on including 14 out of 15 proposed standards. These standards are similar to those in box 1, but they are organized in a different way. Box 2 is also divided into two parts. Part 1 includes standards for studies asking patients about the relevance, comprehensiveness and comprehensibility of the PROM. Part 2 includes standards for studies asking professionals about the relevance and comprehensiveness of the PROM. In the Delphi study, the term ‘experts’ was used, but the steering committee decided afterwards that the term ‘professionals’ is more appropriate because patients are considered the primary experts regarding PROMs.

The only standard on which no consensus was reached in round 1 referred to the required number of professionals in a content validity study. It was argued that diversity is more important. However, others argued that a minimum number of professionals may be needed. In round 2, consensus was reached to use the same standard for the required number of professionals as for the required number of patients (at least 7 for a very good rating). For all standards, consensus was reached on what constitutes a very good, adequate, doubtful, or inadequate rating.

In round 4, standards for asking professionals about the comprehensibility of the PROM were added, based on suggestions from panelists, but during pilot-testing members of the steering committee argued that comprehensibility should be evaluated by patients, not professionals. Therefore, the steering committee decided to remove these standards again. It was also decided to remove a standard on whether problems regarding relevance, comprehensibility, and comprehensiveness were appropriately addressed, because adapting a PROM is not part of the design or analysis of a content validity study.

### Criteria for what constitutes good content validity

In round 1, consensus was reached on including 20 out of 21 proposed criteria, referring to relevance of the items for the construct and target population of interest, appropriate response options and recall period, all key concepts included, and whether the PROM instructions, items, response options, and recall period are understood by the population of interest as intended.

We did not reach consensus on avoidance of cultural issues in the wording of PROM items. It was considered not always possible to anticipate on future translations, nor to avoid cultural issues, and modern psychometric techniques may account for cultural bias. In round 3, we reached consensus on not including this criterion. In round 2 and 3, strong arguments were made against the inclusion of a criterion on appropriate mode of administration because this concerns feasibility rather than content validity. In round 4, consensus was reached to remove this criterion. In rounds 3 and 4, consensus was reached to collapse some criteria, leading to a final set of 10 criteria (Table 4). Consensus was reached to rate each criterion either as sufficient (+), insufficient (−), or indeterminate (?).

**Table 4:**
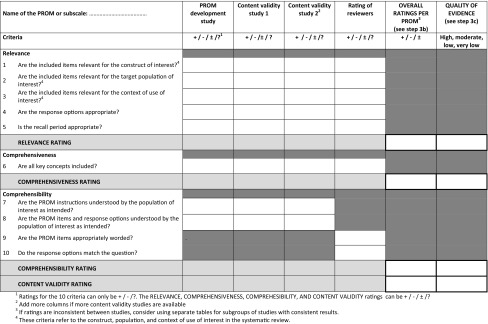
COSMIN criteria and rating system for evaluating the content validity of PROMs

### Rating system for summarizing the evidence on a PROM’s content validity and grading the quality of the evidence in a systematic review

In round 3 and 4, consensus was reached on a rating system for rating the results of the PROM development and available content validity studies against the ten criteria and summarizing all available evidence on a PROM’s content validity and grading the quality of the evidence.

COSMIN considers the measurement properties of each subscale or score of a PROM separately, assuming that each score represents a construct. Therefore, each scale or subscale is rated separately.

The rating system consists of three steps (details are described in the user manual):

First, Table 4 is used to rate the results of the PROM development and available content validity studies against the ten criteria. The reviewers also rate the content of the PROM against these ten criteria. Consensus was reached on how each criterion should be rated. Subsequently, for each study a relevance rating, comprehensiveness rating, comprehensibility rating, and content validity rating are determined by summarizing the five, one, and four criteria for relevance, comprehensiveness, and comprehensibility, respectively. Ratings can be either sufficient (+), insufficient (−), inconsistent (±), or indeterminate (?).

Second, it is determined whether the overall content validity of the PROM is sufficient or insufficient. The focus is here on the PROM, while in the previous step the focus was on the single studies. An overall relevance rating, overall comprehensiveness rating, overall comprehensibility rating, and overall content validity rating are determined for the PROM (second last column Table 4). These ratings will be sufficient (+), insufficient (−), inconsistent (±), or indeterminate (?). If the ratings per study are all sufficient (or all insufficient), the overall rating will also be sufficient (or insufficient). If the ratings are inconsistent between studies, reviewers should explore explanations for the inconsistency (e.g., different study populations or methods). If an explanation is found, overall ratings should be provided within subsets of studies with consistent results. If no explanation is found, the overall rating will be inconsistent (±).

Third, the overall ratings for relevance, comprehensiveness, comprehensibility, and content validity will be accompanied by a grading for the quality of the evidence. This indicates how confident we are that the overall ratings are trustworthy. The evidence can be of high, moderate, low, or very low quality. Using the GRADE factors of risk of bias, inconsistency and indirectness [32], consensus was reached on criteria for high, moderate, low, or very low quality evidence, depending on the type, number and quality of the available studies, the results of the studies, the reviewer’s rating, and the consistency of the results [33]. In grading the quality of evidence, the starting point is always that there is high quality evidence (on a given aspect of content validity). This level of evidence can be downgraded of one or more levels (to moderate, low or very low), if there is (serious or very serious) risk of bias, unexplained inconsistency in results, and/or indirect findings. The thresholds for defining serious or very serious pitfalls can be determined by the review team [13, 32]. The thresholds for defining serious or very serious pitfalls can be determined by the review team.

**Fig. 2:**
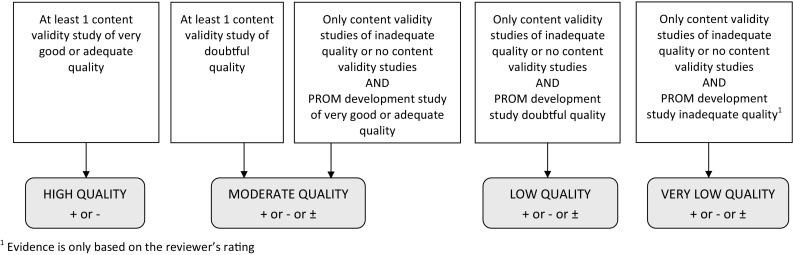
Supplementary flow chart for grading the quality of evidence

In round 3, consensus was reached on using a flow chart for determining the quality of the evidence. After discussion with the chairman of the Dutch GRADE network, a GRADE table of ‘quality assessment criteria’ was developed instead (Table 5). A minimized version of the flow chart (Fig. 2) was kept for additional guidance.

**Table 5:** Grading the quality of evidence on content validity (modified GRADE approach)

Study design	Quality of evidence	Lower if
At least 1 content validity study	High	Risk of bias -1 Serious -2 Very serious
No content validity studies	Moderate	
	Low	Inconsistency -1 Serious -2 Very serious
	Very low	
		Indirectness -1 Serious -2 Very serious

The level of evidence indicates how confident we are that the overall ratings are trustworthy. The starting point is the assumption that the evidence is of high quality. The quality of evidence is subsequently downgraded with one or two levels per factor to moderate, low, or very low when there is risk of bias (low study quality), (unexplained) inconsistency in results, or indirect results

Finally, two changes to the rating system were proposed based on pilot-testing and approved after discussions within the steering committee. First, it was argued that relevance and comprehensiveness of a PROM cannot only be assessed in a qualitative study, but also using a survey. The steering committee decided to consider a survey adequate for evaluating relevance and comprehensiveness if each item of the PROM is evaluated separately. For assessing comprehensibility, a qualitative study is preferred and a doubtful rating will be given if only a survey was performed. Second, the steering committee decided that is was more clear to use criterion 7 and 8 (i.e., PROM instructions, items and response options understood by the population of interest as intended) for rating the results from the PROM development study and content validity studies and to use criteria nine and ten (i.e., items appropriately worded and response options match the question) for rating the content of the PROM by the reviewers.

## Discussion

A consensus-based methodology for rating the content validity of PROMs was developed, including standards for evaluating the quality of PROM development, updated standards for evaluating the quality of content validity studies of existing PROMs, criteria for what constitutes good content validity, and a rating system for summarizing the evidence on a PROM’s content validity and grading the quality of the evidence in systematic reviews of PROMs. The quality of a PROM heavily depends on adequate input from patients in the development of the PROMs and content validity assessment, because patients are the primary experts regarding PROMs.

Some researchers consider statistical analyses on scale and item characteristics also part of content validity assessment. For example, a working group from the PROMIS initiative considers scaling of items part of content validity assessment [15]. However, others, such as the ISPOR taskforce, do not consider such statistical methods part of content validity assessment [4–6, 34, 35]. Within the COSMIN taxonomy, statistical analyses, such as item scaling and factor analysis, are considered to be part of internal consistency and structural validity assessment, and the methodological quality of such studies is evaluated with separate boxes [36]. We agree with Magasi et al. [15] and others that testing the internal structure of a PROM is essential and that it may point to items that are not measuring the same construct. However, we recommend to evaluate the internal structure of the PROM as a next step, after evaluating content validity.

The new COSMIN standards and criteria for content validity are not substantially different from earlier published guidelines, including the original COSMIN standards for content validity, but they are more detailed, standardized, and transparent [3, 20, 21, 23, 24, 37]. Moreover, the COSMIN methodology is unique in that it consists of a scoring method for evaluating (and comparing) the content validity of PROMs in a systematic and transparent way. This is especially relevant for systematic reviews of PROMs. Nevertheless, judgment is still needed, for example, about appropriate qualitative data collection methods and analyses. It was considered not possible to define exactly what is appropriate due to many possible variations in design and analysis of qualitative studies. Moreover, we did not intend to develop a ‘cookbook,’ all of this still boils down to judgment of quality. We recommend that the review team includes reviewers with knowledge of and experience with qualitative research. Furthermore, we recommend that rating is done by two reviewers independently and that consensus-based ratings are reported. The rating system should be further tested in multiple systematic reviews of PROMs to see if it is fit-for-purpose. We strongly encourage reviewers to use the “COSMIN Methodology for assessing the content validity of PROMs—user manual” (http://www.cosmin.nl), which will be regularly updated, if needed. Finally, it is important to ensure that the strength of qualitative methods is not lost in an attempt to standardize the evaluation. Therefore, the COSMIN methodology should be used as guidance, leaving the final judgment to the reviewers based on the available evidence and their methodological and clinical expertise.

The two newly developed COSMIN boxes replace box D (content validity) of the original COSMIN checklist [20]. The other eight boxes of the original COSMIN checklist have also been updated, and together with the boxes for content validity, will form the COSMIN Risk of Bias checklist for PROMs [31]. Assessing content validity is only one step of a systematic review of PROMs. The whole methodology of systematic reviews of PROMs has been described in a recently developed COSMIN guideline [13]. The new methodology for evaluating the content validity of PROMs can contribute to the selection and use of high quality PROMs in research and clinical practice.

## Electronic supplementary material

Below is the link to the electronic supplementary material.

Supplementary material 1 (PDF 323 KB)

Supplementary material 2 (PDF 442 KB)

## References

[1] Mokkink LB, Terwee CB, Patrick DL, Alonso J, Stratford PW, Knol DL, Bouter LM, de Vet HC (2010). The COSMIN study reached international consensus on taxonomy, terminology, and definitions of measurement properties for health-related patient-reported outcomes. Journal of Clinical Epidemiology.

[2] Messick S (1980). Test validity and the ethics of assessment. American Psychologist.

[3] Streiner DL, Norman GR (2008). Health measurement scales. A practical guide to their development and use.

[4] Patrick DL, Burke LB, Gwaltney CJ, Leidy NK, Martin ML, Molsen E, Ring L (2011). Content validity–establishing and reporting the evidence in newly developed patient-reported outcomes (PRO) instruments for medical product evaluation: ISPOR PRO good research practices task force report: Part 1-eliciting concepts for a new PRO instrument. Value Health.

[5] Patrick DL, Burke LB, Gwaltney CJ, Leidy NK, Martin ML, Molsen E, Ring L (2011). Content validity–establishing and reporting the evidence in newly developed patient-reported outcomes (PRO) instruments for medical product evaluation: ISPOR PRO Good Research Practices Task Force report: Part 2-assessing respondent understanding. Value Health.

[6] Brod M, Tesler LE, Christensen TL (2009). Qualitative research and content validity: Developing best practices based on science and experience. Quality of Life Research.

[7] American Educational Research Association, American Psychological Association, National Council on Measurement in Education. (2014). *Standards for educational & psychological testing*. Washington, DC.

[8] U.S.Department of Health and Human ServicesFood and Drug Administration (FDA), Center for Drug Evaluation and Research (CDER), Center for Biologics Evaluation and Research (CBER), Center for Devices and Radiological Health (CDRH). (2009). *Guidance for industry patient-reported outcome measures: Use in medical product development to support labeling claims*. Rockville, MD.

[9] European Medicines Agency. (2005). *Reflection paper on the regulatory guidance for the use of health related quality of life (HRQL) Measures in the evaluation of medicinal products* London.

[10] Sijtsma K (2009). On the use, the misuse, and the very limited usefulness of Cronbach’s Alpha. Psychometrika.

[11] Cortina JM (1993). What is coefficient alpha? An examination of theory and applications. Journal of Applied Psychology.

[12] de Leeuw ED, Hox JJ, Dillman DA (2008). International handbook of survey methodology.

[13] Prinsen, C. A., Mokkink, L. B., Bouter, L. M., Alonso, J., Patrick, D. L., De Vet, H. C. W., & Terwee, C. B. (2017). COSMIN guideline for systematic reviews of outcome measurement instruments. *Quality of Life Research*, Jan 23 [Epub ahead of print]. 10.1007/s11136-018-1798-3 PMC5891568 29435801

[14] Prinsen CA, Vohra S, Rose MR, Boers M, Tugwell P, Clarke M, Williamson PR, Terwee CB (2016). How to select outcome measurement instruments for outcomes included in a “Core Outcome Set”—A practical guideline. Trials.

[15] Magasi S, Ryan G, Revicki D, Lenderking W, Hays RD, Brod M, Snyder C, Boers M, Cella D (2012). Content validity of patient-reported outcome measures: Perspectives from a PROMIS meeting. Quality of Life Research.

[16] Olshansky E, Lakes KD, Vaughan J, Gravem D, Rich JK, David M, Nguyen H, Cooper D (2012). Enhancing the construct and content validity of rating scales for clinical research: Using qualitative methods to develop a rating scale to assess parental perceptions of their role in promoting infant exercise. The International Journal of Education Psychological Assessment.

[17] Vogt DS, King DW, King LA (2004). Focus groups in psychological assessment: Enhancing content validity by consulting members of the target population. Psychological Assessment.

[18] de Vet HCW, Terwee CB, Mokkink LB, Knol DL (2011). Measurement in medicine.

[19] Chiarotto A, Ostelo RW, Boers M, Terwee CB (2017). A systematic review highlights the need to investigate the content validity of patient-reported instruments for physical functioning in low back pain. Journal of Clinical Epidemiology.

[20] Mokkink LB, Terwee CB, Patrick DL, Alonso J, Stratford PW, Knol DL, Bouter LM, de Vet HC (2010). The COSMIN checklist for assessing the methodological quality of studies on measurement properties of health status measurement instruments: An international Delphi study. Quality of Life Research.

[21] Lohr KN, Aaronson NK, Alonso J, Burnam MA, Patrick DL, Perrin EB, Roberts JS (1996). Evaluating quality-of-life and health status instruments: Development of scientific review criteria. Clinical Therapeutics.

[22] Aaronson N, Alonso J, Burnam A, Lohr KN, Patrick DL, Perrin E, Stein RE (2002). Assessing health status and quality-of-life instruments: Attributes and review criteria. Quality of Life Research.

[23] Valderas JM, Ferrer M, Mendivil J, Garin O, Rajmil L, Herdman M, Alonso J (2008). Development of EMPRO: A tool for the standardized assessment of patient-reported outcome measures. Value Health.

[24] Terwee CB, Bot SD, de Boer MR, van der Windt DA, Knol DL, Dekker J, Bouter LM, de Vet HC (2007). Quality criteria were proposed for measurement properties of health status questionnaires. Journal of Clinical Epidemiology.

[25] Reeve BB, Wyrwich KW, Wu AW, Velikova G, Terwee CB, Snyder CF, Schwartz C, Revicki DA, Moinpour CM, McLeod LD, Lyons JC, Lenderking WR, Hinds PS, Hays RD, Greenhalgh J, Gershon R, Feeny D, Fayers PM, Cella D, Brundage M, Ahmed S, Aaronson NK, Butt Z (2013). ISOQOL recommends minimum standards for patient-reported outcome measures used in patient-centered outcomes and comparative effectiveness research. Quality of Life Research.

[26] Guyatt GH, Oxman AD, Vist GE, Kunz R, Falck-Ytter Y, Alonso-Coello P, Schunemann HJ (2008). GRADE: an emerging consensus on rating quality of evidence and strength of recommendations. BMJ.

[27] PROMIS® Instrument Development and Validation Scientific Standards Version 2.0 (2013). Retrieved 29 March 2017, from http://www.healthmeasures.net/explore-measurement-systems/promis/measure-development-research/119-measure-development-research.

[28] Kuper A, Reeves S, Levinson W (2008). An introduction to reading and appraising qualitative research. BMJ.

[29] COSMIN database of systematic reviews of outcome measurement instruments. Retrieved 24 Feb 2015, from http://database.cosmin.nl/.

[30] Terwee CB, Mokkink LB, Knol DL, Ostelo RW, Bouter LM, de Vet HC (2012). Rating the methodological quality in systematic reviews of studies on measurement properties: A scoring system for the COSMIN checklist. Quality of Life Research.

[31] Mokkink, L. B., de Vet, H. C. W., Prinsen, C. A. C., Patrick, D. L., Alonso, J., Bouter, L. M., & Terwee, C. B. (2018). COSMIN risk of bias checklist for assessing the methodological quality of studies on the measurement properties of Patient-Reported Outcome Measures. *Quality of Life Research*, Dec 19 [Epub ahead of print]. 10.1007/s11136-017-1765-4 PMC5891552 29260445

[32] Guyatt G, Oxman AD, Akl EA, Kunz R, Vist G, Brozek J, Norris S, Falck-Ytter Y, Glasziou P, DeBeer H, Jaeschke R, Rind D, Meerpohl J, Dahm P, Schunemann HJ (2011). GRADE guidelines: 1. Introduction-GRADE evidence profiles and summary of findings tables. Journal of Clinical Epidemiology.

[33] Schunemann, H. J., Brozek, J., Guyatt, G. H., & Oxman, A. D. (2013). GRADE Handbook. http://gdt.guidelinedevelopment.org/app/handbook/handbook.html#h.9rdbelsnu4iy.

[34] Leidy NK, Vernon M (2008). Perspectives on patient-reported outcomes: Content validity and qualitative research in a changing clinical trial environment. Pharmacoeconomics.

[35] Rothman M, Burke L, Erickson P, Leidy NK, Patrick DL, Petrie CD (2009). Use of existing patient-reported outcome (PRO) instruments and their modification: the ISPOR good research practices for evaluating and documenting content validity for the use of existing instruments and their modification PRO task force report. Value Health.

[36] Mokkink LB, Terwee CB, Knol DL, Stratford PW, Alonso J, Patrick DL, Bouter LM, de Vet HC (2010). The COSMIN checklist for evaluating the methodological quality of studies on measurement properties: A clarification of its content. BMC Medical Research Methodology.

[37] Zumbo BD, Chan EKH (2014). Validity and validation in social, behavioral, and Health Sciences.

