# Supplementary material for: COSMIN methodology for evaluating the content validity of patient-reported outcome measures: a Delphi study
Source: Qual Life Res. 2018 Mar 17;27(5):1159–70. doi: 10.1007/s11136-018-1829-0 (PMC5891557; doi:10.1007/s11136-018-1829-0)
Supplement: Supplementary file 1 — Supplementary material 1 (PDF 323 KB) [file 11136_2018_1829_MOESM1_ESM.pdf]

COSMIN box 1. Standards for evaluating the quality of PROM development

Part 1a (PROM design) and part 1b (Cognitive interview study or other pilot test) both need to be completed for each PROM because all standards of part 1a and part 1b will be included in the final rating of the quality of the PROM development. However, if a cognitive interview study or other pilot test was not performed, only the first standard in part 1b needs to be completed and the rest of the box can be skipped.

For rating the standards, the "worst score counts" method is used. A total rating for the box can be obtained by taking the lowest rating of any standard in the box. It is also possible to obtain total ratings for different parts of the boxes by taking the lowest rating of any standard of that part of the box.

Box 1. PROM development

1a. PROM design

	General design requirements	**Very good**	**Adequate**	**Doubtful**	**Inadequate**	**Not applicable**
1	Is a clear description provided of the construct to be measured?	Construct clearly described			Construct not clearly described	
2	Is the origin of the construct clear: was a theory, conceptual framework or disease model used or clear rationale provided to define the construct to be measured?	Origin of the construct clear		Origin of the construct not clear		
3	Is a clear description provided of the target population for which the PROM was developed?	Target population clearly described			Target population not clearly described	
4	Is a clear description provided of the context of use	Context of use clearly described		Context of use not clearly described		

5	Was the PROM development study performed in a sample representing the target population for which the PROM was developed?	Study performed in a sample representing the target population	Assumable that the study was performed in a sample representing the target population, but not clearly described	Doubtful whether the study was performed in a sample representing the target population	Study not performed in a sample representing the target population **(SKIP standards 6-12)**	
Concept elicitation (relevance and comprehensiveness)						
6	Was an appropriate qualitative data collection method used to identify relevant items for a new PROM?	Widely recognized or well justified qualitative method used, suitable for the construct and study population	Assumable that the qualitative method was appropriate and suitable for the construct and study population, but not clearly described	Only quantitative (survey) method(s) used or doubtful whether the method was suitable for the construct and study population	Method used not appropriate or not suitable for the construct or study population	
7	Were skilled group moderators/interviewers used?	Skilled group moderators/ interviewers used	Group moderators /interviewers had limited experience or were trained specifically for the study	Not clear if group moderators /interviewers were trained or group moderators /interviewers not trained and no experience		Not applicable
8	Were the group meetings or interviews based on an appropriate topic or interview guide?	Appropriate topic or interview guide	Assumable that the topic or interview guide was appropriate, but not clearly described	Not clear if a topic guide was used or doubtful if topic or interview guide was appropriate or no guide		Not applicable

9	Were the group meetings or interviews recorded and transcribed verbatim?	All group meetings or interviews were recorded and transcribed verbatim	Assumable that all group meetings or interviews were recorded and transcribed verbatim, but not clearly described	Not clear if all group meetings of interviews were recorded and transcribed verbatim or recordings not transcribed verbatim or only notes were made during the group meetings/ interviews	No recording and no notes	Not applicable
10	Was an appropriate approach used to analyse the data?	A widely recognized or well justified approach was used	Assumable that the approach was appropriate, but not clearly described	Not clear what approach was used or doubtful whether the approach was appropriate	Approach not appropriate	
11	Was at least part of the data coded independently?	At least 50% of the data was coded by at least two researchers independently	11-49% of the data was coded by at least two researchers independently	Doubtful if two researchers were involved in the coding or only 1-10% of the data was coded by at least two researchers independently	Only one researcher was involved in coding or no coding	Not applicable
12	Was data collection continued until saturation was reached?	Evidence provided that saturation was reached	Assumable that saturation was reached	Doubtful whether saturation was reached	Evidence suggests that saturation was not reached	Not applicable
13	For quantitative studies (surveys): was the sample size appropriate?	≥100	50-99	30-49	<30	Not applicable

1b. Cognitive interview study or other pilot test		Very good	Adequate	Doubtful	Inadequate	Not applicable
14	Was a cognitive interview study or other pilot test conducted?	YES			NO **(SKIP standards 15-34)**	
General design requirements						
15	Was the cognitive interview study or other pilot test performed in a sample representing the target population?	Study performed in a sample representing the target population	Assumable that the study was performed in a sample representing the target population, but not clearly described	Doubtful whether the study was performed in a sample representing the target population	Study not performed in a sample representing the target population	
Comprehensibility						
16	Were patients asked about the comprehensibility of the PROM?	YES		Not clear **(SKIP standards 17-25)**	NO **(SKIP standards 17-25)**	
17	Were all items tested in their final form?	All items were tested in their final form	Assumable that all items were tested in their final form, but not clearly described	Not clear if all items were tested in their final form	Items were not tested in their final form or items were not re-tested after substantial adjustments	

18	Was an appropriate qualitative method used to assess the comprehensibility of the PROM instructions, items, response options, and recall period?	Widely recognized or well justified qualitative method used	Assumable that the method was appropriate but not clearly described	Only quantitative (survey) method(s) used or doubtful whether the method was appropriate or not clear if patients were asked about the comprehensibility of all items, response options instructions, and recall period or patients not asked about the comprehensibility of the PROM instructions or the recall period	Method used not appropriate or patients not asked about the comprehensibility of all items and response options	
19	Was each item tested in an appropriate number of patients?<br>For qualitative studies<br>For quantitative (survey) studies	 ≥7<br>≥50	 4-6<br>≥30	 <4 or not clear<br><30 or not clear		
20	Were skilled interviewers used?	Skilled group moderators/ interviewers used	Group moderators /interviewers had limited experience or were trained specifically for the study	Not clear if group moderators /interviewers were trained or group moderators /interviewers not trained and no experience		Not applicable

21	Were the interviews based on an appropriate interview guide?	Appropriate topic or interview guide	Assumable that the topic or interview guide was appropriate, but not clearly described	Not clear if a topic guide was used or doubtful if topic or interview guide was appropriate or no guide		Not applicable
22	Were the interviews recorded and transcribed verbatim?	All group meetings or interviews were recorded and transcribed verbatim	Assumable that all group meetings or interviews were recorded and transcribed verbatim, but not clearly described	Not clear if all group meetings or interviews were recorded and transcribed verbatim or recordings not transcribed verbatim or only notes were made during the group meetings/ interviews	No recording and no notes	Not applicable
23	Was an appropriate approach used to analyse the data?	A widely recognized or well justified approach was used	Assumable that the approach was appropriate, but not clearly described	Not clear what approach was used or doubtful whether the approach was appropriate	Approach not appropriate	
24	Were at least two researchers involved in the analysis?	At least two researchers involved in the analysis	Assumable that at least two researchers were involved in the analysis, but not clearly described	Not clear if two researchers were included in the analysis or only one researcher involved in the analysis		

25	Were problems regarding the comprehensibility of the PROM instructions, items, response options, and recall period appropriately addressed by adapting the PROM?	No problems found or problems appropriately addressed and PROM was adapted and re-tested if necessary	Assumable that there were no problems or that problems were appropriately addressed, but not clearly described	Not clear if there were problems or doubtful if problems were appropriately addressed	Problems not appropriately addressed or PROM was adapted but items were not re-tested after substantial adjustments.	Not applicable
Comprehensiveness						
26	Were patients asked about the <u>comprehensiveness</u> of the PROM?	YES		NO or not clear **(SKIP standards 27-35)**		
27	Was the final set of items tested?	The final set of items was tested	Assumable that the final set of items was tested, but not clearly described	Not clear if the final set of items was tested or not the final set of items was tested or the set of items was not re-tested after items were removed or added		
28	Was an appropriate method used for assessing the comprehensiveness of the PROM?	Widely recognized or well justified method used	Assumable that the method was appropriate but not clearly described or only quantitative (survey) method(s) used	Doubtful whether the method was appropriate or method used not appropriate		

29	Was each item tested in an appropriate number of patients?<br>For qualitative studies<br>For quantitative (survey) studies	 ≥7<br>≥50	 4-6<br>≥30	 <4 or not clear<br><30 or not clear		
30	Were skilled interviewers used?	Skilled interviewers used	Interviewers had limited experience or were trained specifically for the study	Not clear if interviewers were trained or interviewers not trained and no experience		Not applicable
31	Were the interviews based on an appropriate interview guide?	Appropriate topic or interview guide	Assumable that the topic or interview guide was appropriate, but not clearly described	Not clear if a topic guide was used or doubtful if topic or interview guide was appropriate or no guide		Not applicable
32	Were the interviews recorded and transcribed verbatim?	All group meetings or interviews were recorded and transcribed verbatim	Assumable that all group meetings or interviews were recorded and transcribed verbatim, but not clearly described	Not clear if all group meetings or interviews were recorded and transcribed verbatim or recordings not transcribed verbatim or only notes were made during the group meetings/ interviews or no recording and no notes		Not applicable

33	Was an appropriate approach used to analyse the data?	A widely recognized or well justified approach was used	Assumable that the approach was appropriate, but not clearly described	Not clear what approach was used or doubtful whether the approach was appropriate or approach not appropriate		
34	Were at least two researchers involved in the analysis?	At least two researchers involved in the analysis	Assumable that at least two researchers were involved in the analysis, but not clearly described	Not clear if two researchers were included in the analysis or only one researcher involved in the analysis		
35	Were problems regarding the <u>comprehensiveness</u> of the PROM appropriately addressed by adapting the PROM?	No problems found or problems appropriately addressed and PROM was adapted and re-tested if necessary	Assumable that there were no problems or that problems were appropriately addressed, but not clearly described	Not clear if there were problems or doubtful if problems were appropriately addressed or PROM was adapted but items were not re-tested after substantial adjustments	Problems not appropriately addressed	Not applicable
