# Supplementary material for: COSMIN methodology for evaluating the content validity of patient-reported outcome measures: a Delphi study
Source: Qual Life Res. 2018 Mar 17;27(5):1159–70. doi: 10.1007/s11136-018-1829-0 (PMC5891557; doi:10.1007/s11136-018-1829-0)
Supplement: Supplementary file 2 — Supplementary material 2 (PDF 442 KB) [file 11136_2018_1829_MOESM2_ESM.pdf]

COSMIN box 2. Standards for evaluating the quality of content validity studies of PROMs

Only those parts of the box need to be completed for which information is available. For example, if a content validity study was not performed in professionals, parts 2d and 2e do not need to be completed. If patients were included in a content validity study, but they were only asked about comprehensibility of the PROM items, sections 2a and 2b do not need to be completed.

For rating the standards, the "worst score counts" method is used. A total score for the box can be obtained by taking the lowest rating of any standard in the box. It is also possible to obtain total ratings for different parts of the boxes by taking the lowest rating of any standard of that part of the box.

Box 2. Content validity

2a. Asking patients about relevance

Design requirements		**Very good**	**Adequate**	**Doubtful**	**Inadequate**	**Not applicable**
1	Was an appropriate method used to ask patients whether each item is <u>relevant</u> for their experience with the condition?	Widely recognized or well justified method used	Only quantitative (survey) method(s) used or assumable that the method was appropriate but not clearly described	Not clear if patients were asked whether <u>each</u> item is relevant or doubtful whether the method was appropriate	Method used not appropriate or patients not asked about the relevance of all items	
2	Was each item tested in an appropriate number of patients?					
	For qualitative studies	≥7	4-6	<4 or not clear		
	For quantitative (survey) studies	≥50	≥30	<30 or not clear		

3	Were skilled group moderators/interviewers used?	Skilled group moderators/ interviewers used	Group moderators /interviewers had limited experience or were trained specifically for the study	Not clear if group moderators /interviewers were trained or group moderators /interviewers not trained and no experience		Not applicable
4	Were the group meetings or interviews based on an appropriate topic or interview guide?	Appropriate topic or interview guide	Assumable that the topic or interview guide was appropriate, but not clearly described	Not clear if a topic guide was used or doubtful if topic or interview guide was appropriate or no guide		Not applicable
5	Were the group meetings or interviews recorded and transcribed verbatim?	All group meetings or interviews were recorded and transcribed verbatim	Assumable that all group meetings or interviews were recorded and transcribed verbatim, but not clearly described	Not clear if all group meetings or interviews were recorded and transcribed verbatim or recordings not transcribed verbatim or only notes were made during the group meetings/ interviews	No recording and no notes	Not applicable

Analyses					
6 Was an appropriate approach used to analyse the data?	A widely recognized or well justified approach was used	Assumable that the approach was appropriate, but not clearly described	Not clear what approach was used or doubtful whether the approach was appropriate	Approach not appropriate	
7 Were at least two researchers involved in the analysis?	At least two researchers involved in the analysis	Assumable that at least two researchers were involved in the analysis, but not clearly described	Not clear if two researchers were included in the analysis or only one researcher involved in the analysis		

2b. Asking patients about comprehensiveness						
Design requirements		**Very good**	**Adequate**	**Doubtful**	**Inadequate**	**Not applicable**
8	Was an appropriate method used for assessing the comprehensiveness of the PROM?	Widely recognized or well justified method used	Only quantitative (survey) method(s) used or assumable that the method was appropriate but not clearly described	Doubtful whether the method was appropriate	Method used not appropriate	
9	Was each item tested in an appropriate number of patients?<br>For qualitative studies<br>For quantitative (survey) studies	 ≥7<br>≥50	 4-6<br>30-49	 <4 or not clear<br><30 or not clear		
10	Were skilled group moderators/interviewers used?	Skilled group moderators/ interviewers used	Group moderators /interviewers had limited experience or were trained specifically for the study	Not clear if group moderators /interviewers were trained or group moderators /interviewers not trained and no experience		Not applicable
11	Were the group meetings or interviews based on an appropriate topic or interview guide?	Appropriate topic or interview guide	Assumable that the topic or interview guide was appropriate, but not clearly described	Not clear if a topic guide was used or doubtful if topic or interview guide was appropriate or no guide		Not applicable

12	Were the group meetings or interviews recorded and transcribed verbatim?	All group meetings or interviews were recorded and transcribed verbatim	Assumable that all group meetings or interviews were recorded and transcribed verbatim, but not clearly described	Not clear if all group meetings or interviews were recorded and transcribed verbatim or recordings not transcribed verbatim or only notes were made during the group meetings/ interviews	No recording and no notes	Not applicable
Analyses						
13	Was an appropriate approach used to analyse the data?	A widely recognized or well justified approach was used	Assumable that the approach was appropriate, but not clearly described	Not clear what approach was used or doubtful whether the approach was appropriate	Approach not appropriate	
14	Were at least two researchers involved in the analysis?	At least two researchers involved in the analysis	Assumable that at least two researchers were involved in the analysis, but not clearly described	Not clear if two researchers were included in the analysis or only one researcher involved in the analysis		

**2c. Asking patients about comprehensibility**					
Design requirements	**Very good**	**Adequate**	**Doubtful**	**Inadequate**	**Not applicable**
15 Was an appropriate qualitative method used for assessing the comprehensibility of the PROM instructions, items, response options, and recall period?	Widely recognized or well justified qualitative method used	Assumable that the method was appropriate but not clearly described	Only quantitative (survey) method(s) used or doubtful whether the method was appropriate or not clear if patients were asked about the comprehensibility of the items, response options or recall period or patients not asked about the comprehensibility of the PROM instructions	Method used not appropriate or patients not asked about the comprehensibility of the items, response options, or recall period	
16 Was each item tested in an appropriate number of patients?					
For qualitative studies	≥7	4-6	<4 or not clear		
For quantitative (survey) studies	≥50	≥30	<30 or not clear		
17 Were skilled group moderators/interviewers used?	Skilled group moderators/ interviewers used	Group moderators /interviewers had limited experience or were trained specifically for the study	Not clear if group moderators /interviewers were trained or group moderators /interviewers not trained and no experience		

18	Were the group meetings or interviews based on an appropriate topic or interview guide?	Appropriate topic or interview guide	Assumable that the topic or interview guide was appropriate, but not clearly described	Not clear if a topic guide was used or doubtful if topic or interview guide was appropriate or no guide		Not applicable
19	Were the group meetings or interviews recorded and transcribed verbatim?	All group meetings or interviews were recorded and transcribed verbatim	Assumable that all group meetings or interviews were recorded and transcribed verbatim, but not clearly described	Not clear if all group meetings or interviews were recorded and transcribed verbatim or recordings not transcribed verbatim or only notes were made during the group meetings/ interviews	No recording and no notes	Not applicable
Analyses						
20	Was an appropriate approach used to analyse the data?	A widely recognized or well justified approach was used	Assumable that the approach was appropriate, but not clearly described	Not clear what approach was used or doubtful whether the approach was appropriate	Approach not appropriate	
21	Were at least two researchers involved in the analysis?	At least two researchers involved in the analysis	Assumable that at least two researchers were involved in the analysis, but not clearly described	Not clear if two researchers were included in the analysis or only one researcher involved in the analysis		

2d. Asking professionals about relevance					
Design requirements	**Very good**	**Adequate**	**Doubtful**	**Inadequate**	**Not applicable**
22 Was an appropriate method used to ask professionals whether each item is relevant for the construct of interest?	Widely recognized or well justified method used	Only quantitative (survey) method(s) used or assumable that the method was appropriate but not clearly described	Not clear if professionals were asked whether each item is relevant or doubtful whether the method was appropriate	Method used not appropriate or professionals not asked about the relevance of all items	
23 Were professionals from all relevant disciplines included?	Professionals from all required disciplines were included	Assumable that professionals from all required disciplines were included, but not clearly described	Doubtful whether professionals from all required disciplines were included or relevant professionals were not included		
24 Was each item tested in an appropriate number of professionals?<br>For qualitative studies<br>For quantitative (survey) studies	 ≥7<br>≥50	 4-6<br>≥30	 <4 or not clear<br><30 or not clear		
Analyses					
25 Was an appropriate approach used to analyse the data?	A widely recognized or well justified approach was used	Assumable that the approach was appropriate, but not clearly described	Not clear what approach was used or doubtful whether the approach was appropriate	Approach not appropriate	

26	Were at least two researchers involved in the analysis?	At least two researchers involved in the analysis	Assumable that at least two researchers were involved in the analysis, but not clearly described	Not clear if two researchers were included in the analysis or only one researcher involved in the analysis		

**2e. Asking professionals about comprehensiveness**						
	Design requirements	**Very good**	**Adequate**	**Doubtful**	**Inadequate**	**Not applicable**
27	Was an appropriate method used for assessing the comprehensiveness of the PROM?	Widely recognized or well justified method used	Only quantitative (survey) method(s) used or assumable that the method was appropriate but not clearly described	Doubtful whether the method was appropriate	Method used not appropriate	
28	Were professionals from all relevant disciplines included?	Professionals from all required disciplines were included	Assumable that professionals from all required disciplines were included, but not clearly described	Doubtful whether professionals from all required disciplines were included or relevant professionals were not included		
29	Was each item tested in an appropriate number of professionals?<br>For qualitative studies<br>For quantitative (survey) studies	 ≥7<br>≥50	 4-6<br>≥30	 <4 or not clear<br><30 or not clear		

Analyses						
30	Was an appropriate approach used to analyse the data?	A widely recognized or well justified approach was used	Assumable that the approach was appropriate, but not clearly described	Not clear what approach was used or doubtful whether the approach was appropriate	Approach not appropriate	
31	Were at least two researchers involved in the analysis?	At least two researchers involved in the analysis	Assumable that at least two researchers were involved in the analysis, but not clearly described	Not clear if two researchers were included in the analysis or only one researcher involved in the analysis		
